# Sequential decision-making in endovascular stroke therapy: benefit and harm of the next retrieval attempt after failed reperfusion

**DOI:** 10.1186/s42466-026-00543-y

**Published:** 2026-10-26

**Authors:** Luca Meucci, Lukas Meyer, Gabriel Broocks, Matthias Bechstein, Christian Thaler, Christian Heitkamp, Laurens Winkelmeier, Vincent Geest, Alexander Heitkamp, Gregor Peter, Felix Schlicht, Caspar Brekenfeld, Máté E. Maros, Maximilian Schell, Maxim Bester, Götz Thomalla, Jens Fiehler, Susanne Gellißen, Fabian Flottmann, Helge C. Kniep

**Affiliations:** 1 Department of Diagnostic and Interventional Neuroradiology, University Medical Center Hamburg-Eppendorf, Martinistraße 52, 20246 Hamburg, Germany; 2 Department of Neuroradiology, HELIOS Medical Center, Campus of MSH Medical School Hamburg, Schwerin, Germany; 3 Department of Biomedical Informatics, Medical Faculty Mannheim, Mannheim, Germany; 4 Department of Neurology, University Medical Center Hamburg-Eppendorf, Hamburg, Germany

## Abstract

**Background:**

After failed reperfusion in endovascular thrombectomy (EVT), interventionalists must outweigh the expected benefit of an additional retrieval attempt with the outcome of the current angiographic result. Framing EVT as an infinite-horizon optimal stopping problem, the present study provides a per-pass evaluation of the expected benefit of EVT continuation versus procedural termination after unsuccessful reperfusion, based on large-scale real-world registry data.

**Methods:**

Patients from the German Stroke Registry – Endovascular Treatment (GSR-ET) between 2015 and 2023 were screened. Inclusion criteria were (1) age ≥ 18 years, (2) anterior circulation LVO, (3) 1–15 retrieval attempts and (4) availability of relevant clinical data. Primary endpoint was the rate of good functional outcome, defined as modified Rankin Scale (mRS) score 0–2 at 90 days. For each retrieval attempt, patients were stratified into successful reperfusion (mTICI 2b–3) and persistent occlusion (mTICI 0–2a). The net expected benefit of EVT continuation, Δ(k), was defined as the difference between the mRS 0–2 rate at pass k + 1, and the mRS 0–2 rate for unsuccessful reperfusion at pass k. A positive Δ(k) indicates that continuation is favored at that decision node k. The pass at which Δ(k) first becomes non-significant identifies the empirical, optimal stopping threshold.

**Results:**

Among 9,495 included patients, successful reperfusion (mTICI 2b–3) was achieved in 8,405 (89%), and 3,428 (36%) achieved good functional outcome at 90 days. Up to six retrieval attempts, Δ(k) was positive and statistically significant (*p* < 0.05), indicating that continuation of EVT was associated with superior expected outcome at each of these decision points. At pass seven, Δ(k) became negative and lost statistical significance (*p* = 0.53), identifying this as the empirical optimal stopping threshold.

**Conclusion:**

Continuation of EVT up to six retrieval attempts confers a significant functional benefit over termination after unsuccessful reperfusion. These findings provide a decision-theoretic basis for individualized intraprocedural decision-making and suggest that functional independence can be achieved beyond conventionally assumed pass thresholds.

**Supplementary Information:**

The online version contains supplementary material available at https://doi.org/10.1186/s42466-026-00543-y.

## Introduction

Endovascular thrombectomy (EVT) is the standard of care in large-vessel occlusion (LVO) acute ischemic stroke (AIS) for selected groups of patients [1–6], with eligibility criteria continually expanding [7, 8]. Within this context, the relationship between retrieval attempts, angiographic reperfusion levels, and clinical outcome remains a topic of scientific and practical relevance with the most favorable outcomes observed after complete reperfusion (modified treatment in cerebral infarction [mTICI] 3) achieved in a single retrieval attempt (“first-pass effect”) [9, 10]. However, persistent occlusions (mTICI 0–2a) are observed in up to 54% of the cases following the first retrieval attempt [11]. Therefore, continuation of the thrombectomy procedure is required in a substantial proportion of cases to achieve satisfactory angiographic results. At the same time, an increasing number of retrieval attempts has been associated with poorer functional outcomes [12, 13], presumably due to the inherent risk of periprocedural complications (i.e. distal embolization, clot migration, dissection or perforation), as well as an increased risk for symptomatic intracranial hemorrhage [14].

Consequently, interventionalists face a dilemma when dealing with persistent occlusions during EVT: While additional retrieval attempts may improve the probability of successful reperfusion and consequently of good clinical outcome [15], they also carry the risk of ultimately resulting in persistent occlusion after multiple attempts, a potentially more detrimental scenario than early termination of the procedure [13].

The EVT procedure is a sequential, time-sensitive decision process: after each retrieval attempt, the interventionalist must evaluate the current angiographic level and determine whether to continue or terminate the procedure. Therefore, from a decision theory standpoint, EVT constitutes an infinite-horizon optimal stopping problem. The procedure is terminated when a stopping rule is satisfied, such as successful reperfusion, the occurrence of a procedural complication, or the judgment that further attempts are futile. Each retrieval attempt constitutes a decision node at which the expected clinical utility of a subsequent retrieval attempt must be weighed against the utility of accepting the current angiographic result.

While several studies have examined functional outcomes in relation to the total number of retrieval attempts and their corresponding reperfusion levels [13, 16], an analysis focusing on sequential infinite-horizon per-pass decision-making in EVT has not been performed yet.

Using real-world data from a large multi-center registry, the present study compares at each pass the expected clinical outcome of an additional retrieval attempt, weighted by the empirical probability of achieving successful reperfusion, against the outcome of procedural termination at the current reperfusion level. The aim is to provide evidence-based support for sequential per-pass intraprocedural decision-making during EVT.

## Methods

### Data availability

The data that support the findings of this study are available upon reasonable request after approval by the German Stroke Registry – Endovascular Treatment (GSR-ET) steering committee.

### Study design and participating centers

The German Stroke Registry – Endovascular Treatment (GSR-ET) is an ongoing, open-label, prospective, multicenter registry of consecutively collected patients undergoing EVT among 25 participating sites in Germany. The main inclusion criteria of GSR-ET are diagnosis of AIS due to LVO, initiation of an endovascular procedure for treatment and age ≥ 18 years, according to national guidelines. There are no exclusion criteria. A detailed description of the GSR-ET study design has been published [17]. The study was approved by the ethics committee of the chamber of physicians at Ludwig-Maximilians University LMU, Munich, as the leading ethics committee, in accordance with the Declaration of Helsinki (ClinicalTrials.gov identifier: NCT03356392, Registration Date: November 22, 2017). Approval by local ethics committees or institutional review boards is obtained for all participating sites according to local regulations. Informed consent for this study was waived after review of the central ethics committee.

### Patients

All patients enrolled in the German Stroke Registry (GSR) between 06/2015 and 12/2023 were screened. Inclusion criteria for the present study were (1) age ≥ 18 years, (2) vessel occlusion in the anterior circulation undergoing EVT, (3) 1 to15 retrieval attempts and (4) availability of relevant clinical data.

### Primary endpoint

The primary endpoint was the rate of good functional outcome, defined as modified Rankin Scale (mRS) score 0–2 at 90 days.

### Clinical data

All clinical parameters are site reported [17], including baseline clinical characteristics, radiological information, procedural and outcome parameters. Reperfusion success was assessed by the treating interventionalist using the mTICI score.

### Statistics

Standard descriptive statistics were used for all reported parameters. Continuous variables were reported as median and interquartile range (IQR). Categorical variables were displayed by absolute frequencies and percentages. Categorical variables were compared using the Chi-squared test for independence, while the non-parametric Mann-Whitney U test was used for variance analyses on numeric variables.

To model sequential intraprocedural decision-making, we formalized EVT as an infinite-horizon optimal stopping problem in which the interventionalist determines at each pass, or decision node, k whether to continue or terminate the procedure. The optimal action at each decision node is determined by whether the expected utility (eU) of attempting an additional retrieval exceeds the utility (U) of accepting the current reperfusion level. For each attempt k, three outcome entities were estimated directly from registry data, using the probability of good functional outcome (modified Rankin Scale [mRS] 0–2 at 90 days) as the utility measure:

- U_success(k): observed mRS 0–2 rate among patients achieving successful reperfusion (mTICI 2b–3) at pass k.
- U_stop(k): observed mRS 0–2 rate among patients with persistent occlusion (mTICI 0–2a) after pass k, representing the utility of procedural termination at the current state.
- eU_continue(k): observed mRS 0–2 rate among all patients who underwent pass k + 1, irrespective of the final angiographic result, implicitly weighting the outcome conditional on successful recanalization by p(k) and the outcome conditional on persistent occlusion by [1 − p(k)], thereby yielding an estimate of the expected utility of continuation without requiring these components to be modeled separately.

The success probability p(k) (likelihood of achieving mTICI 2b–3 on pass k) was estimated as the proportion of patients reaching mTICI 2b–3 among all those who underwent pass k. The primary estimand was the net expected benefit of continuation at each pass, defined as:

$$\Delta \mathrm{(k)} = \mathrm{eU}\_\mathrm{continue(k)} - \mathrm{U}\_\mathrm{stop(k)}$$

A positive Δ(k) indicates that proceeding with an additional retrieval attempt is associated with superior expected outcome compared with terminating the procedure at the current reperfusion level. A negative or non-significant Δ(k) identifies the empirical optimal stopping threshold.

eU_continue(k) is estimated from patients who proceeded to pass k + 1 in real-world clinical practice, with their 90-day outcomes integrating all downstream decisions and passes that followed. The approach constitutes an empirical realization of the Bellman continuation value, the recursive quantity that, in the formal optimal stopping framework, encodes the expected utility of all future decision nodes beyond pass k. Between-group differences in the probability of favorable functional outcome were assessed using a two-sample Welch t-test comparing individual-level mRS 0–2 outcomes between patients who underwent pass k + 1 (eU_continue) and patients for whom the procedure was terminated at pass k with persistent occlusion (U_stop).

Statistical analyses were conducted using R version 4.1.3. Statistical significance level was set at *p* < 0.05.

## Results

### Baseline demographics and clinical data at baseline and follow-up

A total of 9,495 patients fulfilled the inclusion criteria. The median age of the overall cohort was 77 years (IQR, 66–83), and 5,083 patients (54%) were women. Hypertension occurred in 7,238 patients (76%), diabetes mellitus in 2,151 (23%), dyslipidemia in 3,989 (42%), and atrial fibrillation in 4,165 (44%). Median baseline NIHSS score was 14 (IQR, 9–18), and median ASPECTS was 9 (IQR, 7–10). Intravenous thrombolysis was administered in 4,477 (47%) patients. The median number of thrombectomy passes was 2 (IQR, 1–3), and successful reperfusion (mTICI 2b–3) was achieved in 8,405 (89%) patients. Adverse events were uncommon, with vasospasm occurring in 367 (4%) patients, clot migration or embolization in 382 (4%), vessel dissection or perforation in 260 (3%), and intracranial hemorrhage in 345 (4%). At 90 days, the median mRS for the overall cohort was 3 (IQR, 2–6). A complete overview of the defining demographics, clinical and radiological information is shown in Table 1. A complete inclusion flow chart is shown in Supplemental Fig. 1.

**Table 1:** Defining baseline demographics, clinical and radiological information stratified by good functional outcome at 90 days

	90d mRS 0–2	90d mRS 3–6	All	*p*-value
	(*N* = 3,428)	(*N* = 6,067)	(*N* = 9,495)	
***Demographics***				
Age, Median (IQR)	70 (60, 78),	80.0 (71, 85),	77.0 (66, 83),	< 0.01†
	*N* = 3,428	*N* = 6,067	*N* = 9,495	
Sex (w)	1,578/3,428 (46%)	3,505/6,067 (58%)	5,083/9,495 (54%)	< 0.01‡
***Baseline clinical information***				
Hypertension	2,376/3,428 (69%)	4,862/6,067 (80%)	7,238/9,495 (76%)	< 0.01‡
Diabetes mellitus	592/3,428 (17%)	1,559/6,067 (26%)	2,151/9,495 (23%)	< 0.01‡
Dyslipidemia	1,442/3,428 (42%)	2,547/6,067 (42%)	3,989/9,495 (42%)	0.20‡
Atrial fibrillation	1,200/3,428 (35%)	2,965/6,067 (49%)	4,165/9,495 (44%)	< 0.01‡
Baseline NIHSS, Median (IQR)	11 (6, 15),	16 (12, 19),	14 (9, 18),	< 0.01†
	*N* = 3,400	*N* = 5,982	*N* = 9,382	
Baseline ASPECTS, Median (IQR)	9 (8, 10),	8 (7, 10),	9 (7, 10),	< 0.01†
	*N* = 3,157	*N* = 5,533	*N* = 8,690	
I.v. thrombolysis	1,934/3,423 (56%)	2,543/6,050 (42%)	4,477/9,473 (47%)	< 0.01‡
***Occluded vessel***				
Intracranial ICA	581/3,428 (17%)	1,576/6,067 (26%)	2,157/9,495 (23%)	< 0.01‡
MCA M1	1,738/3,428 (51%)	3,120/6,067 (51%)	4,858/9,495 (51%)	0.50‡
MCA M2	849/3,428 (25%)	1,078/6,067 (18%)	1,927/9,495 (20%)	< 0.01‡
***Periprocedural information***				
Number of passes, Median (IQR)	1 (1, 2),	2 (1, 3),	2 (1, 3),	< 0.01†
	*N* = 3,428	*N* = 6,067	*N* = 9,495	
mTICI 2b–3	3,288/3,428 (96%)	5,117/6,067 (84%)	8,405/9,495 (89%)	< 0.01‡
**Adverse events**				
Vasospasm	153/3,428 (4%)	214/6,067 (4%)	367/9,495 (4%)	0.02‡
Clot migration/embolization	90/3,428 (3%)	292/6,067 (5%)	382/9,495 (4%)	< 0.01‡
Dissection/perforation	68/3,428 (2%)	192/6,067 (3%)	260/9,495 (3%)	< 0.01‡
ICH	68/3,428 (2%)	277/6,067 (5%)	345/9,495 (4%)	< 0.01‡
***Follow-up***				
90d mRS, Median (IQR)	1 (0, 2),	5 (4, 6),	3 (2, 6),	< 0.01†
	*N* = 3,428	*N* = 6,067	*N* = 9,495	

†: Mann-Whitney-U test for numeric variables

‡: Chi-square test for categorical variables

NIHSS, National Institutes of Health Stroke Scale; ASPECTS, Alberta Stroke Program Early CT Score; ICA, Internal Carotid Artery; MCA, Middle Cerebral Artery; mTICI, modified Thrombolysis in Cerebral Infarction; ICH, Intracranial Hemorrhage; mRS, modified Rankin Scale

### Per pass functional outcomes if EVT is terminated

After the first retrieval attempt, successful reperfusion (mTICI 2b–3) with subsequent termination of the procedure was achieved in 4,136 patients (43.6%), of whom 44.4% had a good functional outcome (mRS 0–2) at 90 days. In 198 cases (2.1%), the procedure was terminated after the first attempt despite unsuccessful reperfusion (mTICI 0–2a), with 22.2% achieving mRS 0–2 at 90 days (Table 2). With increasing numbers of retrieval attempts, the probability of successful reperfusion (mTICI 2b–3) declined from 39.5% for the second pass to 24.4% for ≥ 7 retrieval attempts. Accordingly, the probability of a good functional outcome at 90 days decreased from 39.0% after the second pass to 31.7% after ≥ 7 passes for cases with successful reperfusion (mTICI 2b–3), and from 17.7% to 5.5% in cases in which the procedure was terminated despite persistent occlusion (mTICI 0–2a) (Table 2).

### Expected functional outcomes for continued EVT retrieval attempts

After the first retrieval attempt, the EVT procedure was continued in 5,161 cases (54.4%). Among these patients, the expected rate of good functional outcome (mRS 0–2 at 90 days) for a subsequent retrieval attempt, accounting for the observed probabilities of successful reperfusion (mTICI 2b–3), persistent occlusion (mTICI 0–2a), early termination, and further continuation, was 30.0%. This rate was significantly higher than the observed 90-day mRS 0–2 rate among patients in whom the procedure was terminated after the first attempt despite unsuccessful reperfusion (mTICI 0–2a) (*p* = 0.01).

This pattern remained consistent for up to six retrieval attempts, with the expected 90-day mRS 0–2 rates for a subsequent retrieval attempt and continued procedure being significantly higher (*p* < 0.05) than the observed rates following early termination of the procedure at the current attempt despite unsuccessful reperfusion (mTICI 0–2a). No statistically significant difference was observed at ≥ 7 retrieval attempts, where the 90-day mRS 0–2 rate after unsuccessful reperfusion (mTICI 0–2a) at the current attempt was 5.5%, compared with an expected rate of 7.9% for subsequent attempts (≥ 8) across all reperfusion grades (*p* = 0.53).

**Fig. 1:**
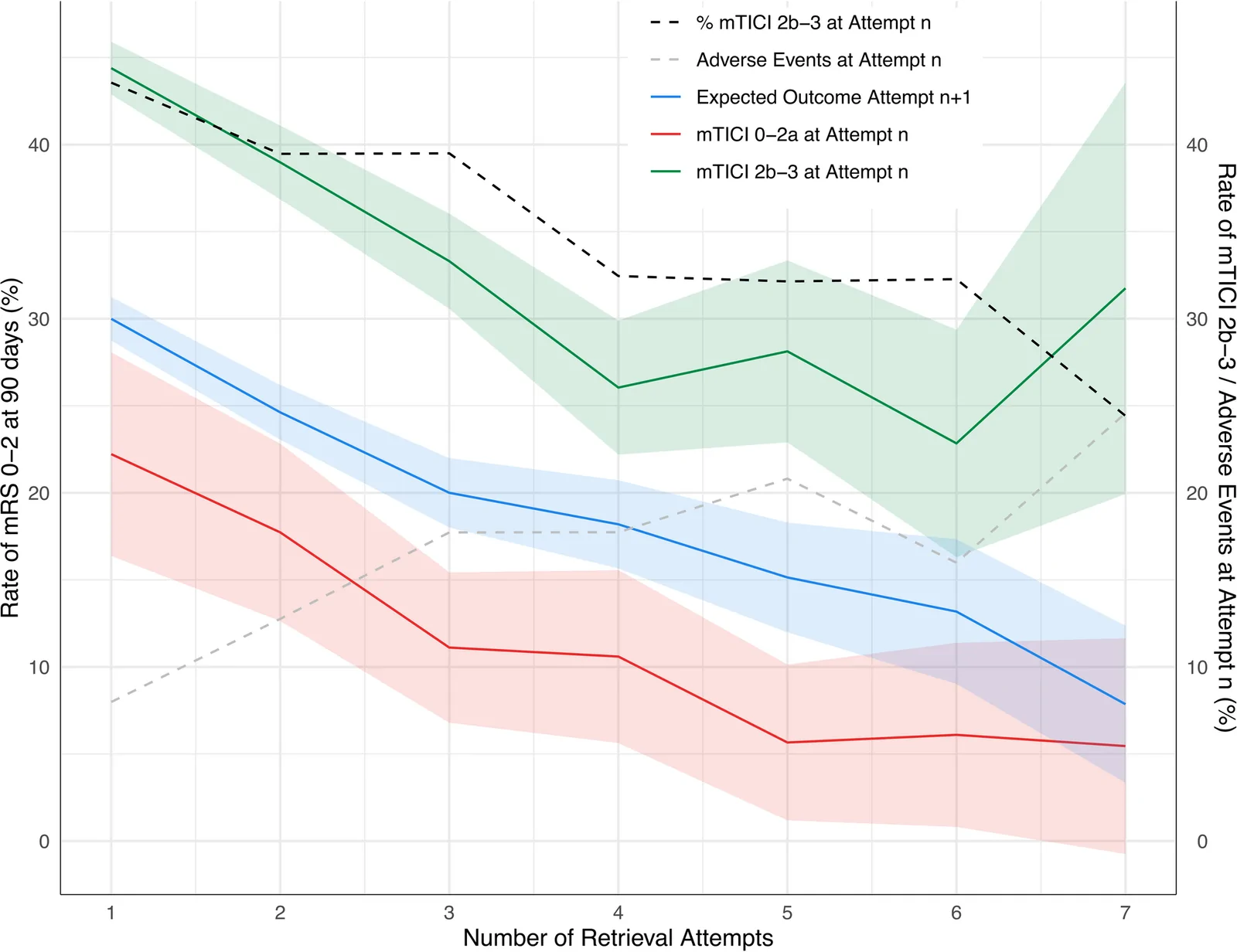
Rates of good functional outcome (mRS 0–2) per retrieval attempt for successful reperfusion (mTICI 2b–3), unsuccessful reperfusion (mTICI 0–2a) and for the subsequent retrieval attempt across all reperfusion levels. Rates of procedure-related adverse events (vasospasm, clot migration/embolization, dissection/perforation, intracranial hemorrhage) are reported for each retrieval attempt. mTICI, modified Thrombolysis in Cerebral Infarction; mRS, modified Rankin scale

The rate of adverse events was lowest among patients undergoing a single retrieval attempt (8.0%) and highest among those undergoing ≥ 7 retrieval attempts (24.6%) as displayed in Fig. 1 and Supplemental Table 1.

**Table 2:** Descriptive statistics of the rate of good functional outcome mRS 0–2 at 90 days for successful reperfusion (mTICI 2b–3), unsuccessful reperfusion (mTICI 0–2a) and the next retrieval attempts across all reperfusion levels

Current Attempt											Expected Value Next Attempt				
mTICI 2b–3 [U_success (k)]						mTICI 0–2a [U_stop(k)]					[eU_continue(k)]				
90d mRS 0–2 (%),95% CI						90d mRS 0–2 (%), 95% CI					90d mRS 0–2 (%), 95% CI				
Pass	Cases	%	mean	low	high	Cases	%	mean	low	high	Cases	mean	low	high	*p*-value
1	4,136	43.6	44.4	42.9	45.9	198	2.1	22.2	16.4	28.1	5,161	30.0	28.7	31.2	0.01
2	2,037	39.5	39.0	36.9	41.1	220	4.3	17.7	12.6	22.8	2,904	24.6	23.1	26.2	0.01
3	1,147	39.5	33.3	30.6	36.0	207	7.1	11.1	6.8	15.4	1,550	20.0	18.0	22.0	< 0.01
4	503	32.5	26.0	22.2	29.9	151	9.7	10.6	5.6	15.6	896	18.2	15.7	20.7	< 0.01
5	288	32.1	28.1	22.9	33.3	106	11.8	5.7	1.2	10.1	502	15.1	12.0	18.3	< 0.01
6	162	32.3	22.8	16.3	29.4	82	16.3	6.1	0.8	11.4	258	13.2	9.0	17.3	0.04
7	63	24.4	31.7	19.9	43.6	55	21.3	5.5	-0.7	11.7	140	7.9	3.3	12.4	0.53

Comparative statistics compare the outcome of the next retrieval attempt with early stopping after unsuccessful reperfusion (mTICI 0–2a). mTICI, modified Thrombolysis in Cerebral Infarction; mRS, modified Rankin scale

## Discussion

This retrospective observational study, based on large-scale multicenter registry data from routine clinical practice, evaluated sequential per-pass decision-making during EVT by formalizing the intraprocedural decision of continuation as an infinite-horizon optimal stopping problem. At each pass, the net expected benefit of continuation was estimated as the difference between the probability-weighted expected utility of an additional retrieval attempt and the utility of procedural termination at the current angiographic state. Δ(k) was positive and statistically significant through pass six, indicating that continuation was associated with superior expected functional outcome at each of these decision nodes.

Several studies over recent years have examined the influence of the number of retrieval attempts and the corresponding reperfusion levels on clinical outcomes in anterior circulation AIS [11–13, 18–21], predominantly based on smaller and often single-center cohorts. Critically, prior analyses evaluated outcomes stratified by total pass count and final reperfusion grade, a retrospective characterization of procedural results that does not map onto the forward-looking, state-dependent decision an interventionalist must make in real time. The present analysis differs fundamentally in its design: rather than asking what outcomes were observed at a given pass, it asks whether the expected outcome of proceeding exceeds the expected outcome of stopping at each individual decision node. This anterograde, per-pass framing directly approximates the sequential decision structure of EVT in routine clinical practice and provides a formally grounded basis for intraprocedural decision support.

In most patients, the EVT procedure is terminated after only a few retrieval attempts. Many studies investigating the relationship between the number of retrieval attempts, angiographic success, and clinical outcome have been limited to single-center case series [20, 22], or relatively small patient cohorts [23]. Consequently, subgroups with higher numbers of retrieval attempts were often numerically small, limiting the ability to reliably assess functional outcomes stratified by the number of retrieval attempts and reducing the statistical power of these analyses. The present study included comparatively large numbers of patients in whom EVT was terminated after five (*n* = 394), six (*n* = 244), and equal or more than seven (*n* = 258) retrieval attempt,as well as a substantially high number of patients undergoing up to four attempts (*n* = 8,599). This large sample size, particularly in “higher attempt number” subgroups, strengthens the statistical power of the analysis and allows for more robust and reliable conclusions.

In our analysis, unsuccessful reperfusion after up to six retrieval attempts was associated with significantly lower rates of good functional outcome compared with the expected outcome of subsequent retrieval attempts across all reperfusion grades. At ≥ 7 retrieval attempts, unsuccessful reperfusion was still associated with numerically lower rates of good functional outcome (90 days mRS 0–2: 5.5%) compared with the expected outcome of subsequent retrieval attempts (90 days mRS 0–2: 7.9%). While statistical significance was not observed (*p* = 0.53), the analysis was based on a sample size of 195 patients, limiting statistical power. However, the overlapping confidence intervals for good functional outcome between unsuccessful reperfusion (mTICI 0–2a) at the sixth attempt and the expected outcome of the subsequent retrieval attempts across all reperfusion grades (Fig. 1) may already indicate a diminishing association between good functional outcome and continuation of EVT.

García et al. reported a decline in clinical outcomes among patients achieving complete reperfusion (mTICI 3) beyond three retrieval attempts in a single-center retrospective cohort of 459 patients [20]. Similarly, other studies have demonstrated decreasing rates of complete reperfusion and suggested that recanalization may be futile beyond the fourth or fifth retrieval attempts [22, 23]. Inversely, Uchida et al. demonstrated that patients achieving successful reperfusion (mTICI 2b–3) with 5, 6 and ≥ 7 retrieval attempts had significantly better clinical outcomes compared to unsuccessful reperfusion (mTICI 0–2a) with ≥ 5 retrieval attempts in a cohort of 407 patients [15]. The latter finding aligns with the results of the present study, suggesting a potential benefit of EVT even after a higher number of retrieval attempts.

Overall, an increasing number of retrieval attempts (k) was associated with lower rates of successful reperfusion (mTICI 2b–3) and lower rates of good functional outcome (mRS 0–2 at 90 days), consistent with previous reports [13, 20]. However, successful reperfusion (mTICI 2b–3) rates after five or more retrieval attempts ranged from 24.4% (for ≥ 7 retrieval attempts) up to 32% (for 5 and 6 retrieval attempts), which were higher than previous reports in the literature [12, 23]. Among these, the rate of good functional outcome (mRS 0–2) was above 22% (28.1% for k = 5; 22.8% for k = 6 and 31.7% for k ≥ 7). Uchida et al. showed comparable rates of 90-days mRS 0–2 for successful reperfusion (mTICI 2b–3) after 5 (28%) and 6 retrieval attempts (19%). However, the rate of patients achieving good clinical outcome following successful reperfusion (mTICI 2b–3) after ≥ 7 retrieval attempts was numerically higher in our cohort (11% vs. 32%).

These findings indicate that, although the rate of futile recanalization increases with a rising number of retrieval attempts, good clinical outcome can still be achieved after a high number of passes in a substantial proportion of patients. In this context, the pursuit of a strict pass-number cut-off beyond which EVT is considered futile may be misleading. What appears consistent across all retrieval attempts is the association between unsuccessful reperfusion (mTICI 0–2a) and unfavorable clinical outcome. Among patients with unsuccessful reperfusion (mTICI 0–2a), an increasing number of retrieval attempts was associated with progressively lower rates of good functional outcome (mRS 0–2 at 90 days), declining from 22.2% after one retrieval attempt to 5.5% in the group with ≥ 7 attempts.

The higher frequency of adverse events observed with increasing numbers of retrieval attempts may have contributed to both procedure termination and lower rates of favourable functional outcomes among patients undergoing multiple retrieval attempts.

Finally, two observations warrant careful interpretation. First, the expected rate of good functional outcome associated with continuation beyond pass three (20.0%) was numerically lower than the observed outcome among patients with persistent occlusion after a single retrieval attempt (22.2%). This might suggest that early termination after the first pass is preferable to late continuation, implying a maximum of three passes as a potential pass threshold. However, this interpretation conflates two fundamentally distinct quantities and is incompatible with the sequential decision structure of EVT. The infinite-horizon optimal stopping framework evaluates an exclusively anterograde decision at each node: given the current reperfusion level at pass k, does the expected utility of proceeding to pass k + 1 exceed the utility of stopping now? This question is answered by Δ(k), the net expected benefit of the next pass relative to the current state, and Δ(k) was positive and significant at every pass from one through six. The comparison between the expected outcome at pass three and the stopping outcome at pass one is therefore not a decision-relevant quantity, since no patient at pass three has the option of returning to pass one.

Second, patients with baseline characteristics associated with poorer functional outcomes after large-vessel occlusion stroke, including higher admission NIHSS scores, lower ASPECTS, poor collateral status, and older age, may have been more likely to undergo earlier termination of thrombectomy if late reperfusion was perceived as unlikely to provide meaningful clinical benefit. Moreover, procedure-related factors, such as unfavourable clot characteristics, challenging vascular anatomy, or device performance, may also have influenced the decision to terminate the procedure. Consequently, patients undergoing a greater number of retrieval attempts may have differed systematically from those in whom thrombectomy was terminated after fewer attempts, introducing potential selection bias and confounding by indication. However, because the reasons for procedure termination were not documented and the present analysis was not adjusted for potential confounders, the extent of these potential biases cannot be derived from our data.

### Limitations

Our study has some limitations. First, we conducted a complete-case analysis, including only cases with all required data points, which may have introduced bias by excluding patients with missing data (including those lost to follow-up) and could reduce the generalizability of our findings. Second, statistical analysis was not adjusted for possible confounding variables influencing clinical outcome in AIS (i.e. age, baseline ASPECTS, baseline NIHSS score, site of vessel occlusion) and possibly introducing bias as patients with a higher number of retrieval attempts may differ from those were interventionalists decided to abort the procedure. Third, the GSR-ET did not capture the specific reasons for procedure termination despite persistent occlusion. Therefore, we could not distinguish between termination due to procedural complications, or other operator- or procedure-related considerations. For example, treatment-related adverse events may have precluded further attempts in individual cases. Fourth, the number of cases with seven or more retrieval attempts was relatively low (*n* = 258), limiting the statistical power of this comparison. Finally, the GSR-ET does not provide information on the modality of 90-day mRS assessment, introducing some potential variability across participating centers within the registry.

## Conclusion

Framing EVT as an infinite-horizon optimal stopping problem, this sequential per-pass analysis of a large-scale multicenter real-world cohort shows that continuation of EVT is associated with a significant net expected functional benefit over procedural termination after unsuccessful reperfusion at each decision node through pass six. At pass seven, the net expected benefit became non-significant, suggesting a possible empirical optimal stopping threshold. These findings provide a decision-theoretic basis for intraprocedural judgment, argue against the imposition of rigid pass-number cut-offs, and suggest that the option value of an additional retrieval attempt remains positive well beyond conventionally assumed procedural limits, supporting individualized, evidence-based decision-making at each step of EVT.

## Supplementary material

Supplementary material 1.

## Data Availability

No datasets were generated or analysed during the current study.

## References

[1] Berkhemer, O. A., Fransen, P. S., Beumer, D., et al. (2015). A randomized trial of intraarterial treatment for acute ischemic stroke. *New England Journal Of Medicine*, *372*(1), 11–20. 10.1056/NEJMoa1411587 25517348

[2] Jovin, T. G., Chamorro, A., Cobo, E., et al. (2015). Thrombectomy within 8 hours after symptom onset in ischemic stroke. *New England Journal Of Medicine*, *372*(24), 2296–2306. 10.1056/NEJMoa1503780 25882510

[3] Saver, J. L., Goyal, M., Bonafe, A., et al. (2015). Stent-retriever thrombectomy after intravenous t-PA vs. t-PA alone in stroke. *New England Journal Of Medicine*, *372*(24), 2285–2295. 10.1056/NEJMoa1415061 25882376

[4] Goyal, M., Demchuk, A. M., Menon, B. K., et al. (2015). Randomized assessment of rapid endovascular treatment of ischemic stroke. *New England Journal Of Medicine*, *372*(11), 1019–1030. 10.1056/NEJMoa1414905 25671798

[5] Campbell, B. C., Mitchell, P. J., Kleinig, T. J., et al. (2015). Endovascular therapy for ischemic stroke with perfusion-imaging selection. *New England Journal Of Medicine*, *372*(11), 1009–1018. 10.1056/NEJMoa1414792 25671797

[6] Ungerer, M. N., Bartig, D., Richter, D., et al. (2024). The evolution of acute stroke care in Germany from 2019 to 2021: analysis of nation-wide administrative datasets. *Neurol Res Pract*, *6*(1), 4. 10.1186/s42466-023-00297-x PMC10782681 38200611

[7] Bendszus, M., Fiehler, J., Subtil, F., et al. (2023). Endovascular thrombectomy for acute ischaemic stroke with established large infarct: multicentre, open-label, randomised trial. *Lancet*, *402*(10414), 1753–1763. 10.1016/S0140-6736(23)02032-9 37837989

[8] Beckonert, N. M., Weller, J. M., Alegiani, A. C., et al. (2024). Endovascular treatment of primary M3 occlusion stroke in clinical practice: analysis of the German Stroke Registry. *Neurol Res Pract*, *6*(1), 36. 10.1186/s42466-024-00330-7 PMC11256396 39020409

[9] Zaidat, O. O., Castonguay, A. C., Linfante, I., et al. (2018). First Pass Effect: A New Measure for Stroke Thrombectomy Devices. *Stroke*, *49*(3), 660–666. 10.1161/STROKEAHA.117.020315 29459390

[10] Nikoubashman, O., Dekeyzer, S., Riabikin, A. (2019). *True First-Pass Effect Stroke*. ;50(8):2140–2146. 10.1161/STROKEAHA.119.025148 31216965

[11] Flottmann, F., Leischner, H., Broocks, G., et al. (2018). Recanalization Rate per Retrieval Attempt in Mechanical Thrombectomy for Acute Ischemic Stroke. *Stroke*, *49*(10), 2523–2525. 10.1161/STROKEAHA.118.022737 30355115

[12] Flottmann, F., Brekenfeld, C., Broocks, G., et al. (2021). Good Clinical Outcome Decreases With Number of Retrieval Attempts in Stroke Thrombectomy: Beyond the First-Pass Effect. *Stroke*, *52*(2), 482–490. 10.1161/STROKEAHA.120.029830 PMC7834657 33467875

[13] Flottmann, F., van Horn, N., Maros, M. E., et al. (2022). More Retrieval Attempts are Associated with Poorer Functional Outcome After Unsuccessful Thrombectomy. *Clinical Neuroradiology*, *32*(2), 361–368. 10.1007/s00062-021-01054-w PMC9187527 34236443

[14] Maros, M. E., Brekenfeld, C., Broocks, G., et al. (2021). Number of Retrieval Attempts Rather Than Procedure Time Is Associated With Risk of Symptomatic Intracranial Hemorrhage. *Stroke*, *52*(5), 1580–1588. 10.1161/STROKEAHA.120.031242 PMC8078115 33813864

[15] Uchida, K., Sowlat, M. M., Matsukawa, H., et al. (2024). Impact of Successful Recanalization and Clinical Outcomes of Patients With Acute Ischemic Stroke with 5 or More Thrombectomy Passes. *Stroke Vasc Interv Neurol*, *4*(1), e001107. 10.1161/SVIN.123.001107 PMC12778462 41586058

[16] Meucci, L., Meyer, L., Broocks, G., et al. (2025). Impact of device pass count and reperfusion levels in anterior circulation stroke: differential effects based on large-scale data from clinical practice. *Journal Of Neurology*, *272*(10), 682. 10.1007/s00415-025-13417-1 41065870

[17] Alegiani, A. C., Dorn, F., Herzberg, M., et al. (2019). Systematic evaluation of stroke thrombectomy in clinical practice: The German Stroke Registry Endovascular Treatment. *International Journal Of Stroke : Official Journal Of The International Stroke Society*, *14*(4), 372–380. 10.1177/1747493018806199 30346260

[18] Flottmann, F., van Horn, N., Maros, M. E., et al. (2022). Early TICI 2b or Late TICI 3-Is Perfect the Enemy of Good? *Clinical Neuroradiology*, *32*(2), 353–360. 10.1007/s00062-021-01048-8 PMC9187567 34191040

[19] Kniep, H. C., Meyer, L., Broocks, G., et al. (2025). Early stopping versus continued retrievals after failed recanalization: associated factors and implications for outcome. *J Neurointerv Surg*, *17*(11), 1207–1215. 10.1136/jnis-2024-022157 39332899

[20] Garcia-Tornel, A., Requena, M., Rubiera, M. (2019). *When to Stop Stroke*. ;50(7):1781–1788. 10.1161/STROKEAHA.119.025088 31177974

[21] Winkelmeier, L., Faizy, T. D., Broocks, G., et al. (2023). Association Between Recanalization Attempts and Functional Outcome After Thrombectomy for Large Ischemic Stroke. *Stroke*, *54*(9), 2304–2312. 10.1161/STROKEAHA.123.042794 PMC10464881 37492970

[22] Seker, F., Pfaff, J., Wolf, M., et al. (2017). Correlation of Thrombectomy Maneuver Count with Recanalization Success and Clinical Outcome in Patients with Ischemic Stroke. *Ajnr. American Journal Of Neuroradiology*, *38*(7), 1368–1371. 10.3174/ajnr.A5212 PMC7959895 28473346

[23] Baek, J. H., Kim, B. M., Heo, J. H., et al. (2018). Number of Stent Retriever Passes Associated With Futile Recanalization in Acute Stroke. *Stroke*, *49*(9), 2088–2095. 10.1161/STROKEAHA.118.021320 30354993

