# Supplementary material for: Sequential decision-making in endovascular stroke therapy: benefit and harm of the next retrieval attempt after failed reperfusion
Source: Neurol Res Pract. 2026 Oct 26;8(1):88. doi: 10.1186/s42466-026-00543-y (PMC13711950; doi:10.1186/s42466-026-00543-y)
Supplement: Supplementary file 1 [file 42466_2026_543_MOESM1_ESM.docx]

**SUPPLEMENTAL MATERIAL**

**Supplemental Figure 1** Patients inclusion flow chart. GSR-ET, German Stroke Registriy-Endovascular Treatment; mRS, modified Rankin scale; EVT, Endovascular Thrombectomy; BA, Basilar Artery; VA, Vertebral Artery; PCA, Posterior Cerebral Artery; ACA, Anterior Cerebral Artery; ICA, Internal Cerebral Artery; mTICI, modified Thrombolysis in Cerebral Infarction.

**Supplemental Table 1** Descriptive statistics of the rate of adverse events and of good functional outcome mRS 0-2 at 90 days for successful reperfusion (mTICI 2b-3) and unsuccessful reperfusion (mTICI 0-2a) for each retrieval attempt. The adverse events included included vasospasm, clot migration/embolization, dissection/perforation, and intracranial hemorrhage.

|  | **Adverse events** |  | **mTICI 2b-3** | | |  | **mTICI 0-2a** | | |
| --- | --- | --- | --- | --- | --- | --- | --- | --- | --- |
| Pass | % |  | Cases | % | mRS 0-2 |  | Cases | % | mRS 0-2 |
| 1 | 8.0 |  | 4,136 | 43.6 | 44.4 |  | 198 | 2.1 | 22.2 |
| 2 | 12.8 |  | 2,037 | 39.5 | 39.0 |  | 220 | 4.3 | 17.7 |
| 3 | 17.7 |  | 1,147 | 39.5 | 33.3 |  | 207 | 7.1 | 11.1 |
| 4 | 17.7 |  | 503 | 32.5 | 26.0 |  | 151 | 9.7 | 10.6 |
| 5 | 20.8 |  | 288 | 32.1 | 28.1 |  | 106 | 11.8 | 5.7 |
| 6 | 16.0 |  | 162 | 32.3 | 22.8 |  | 82 | 16.3 | 6.1 |
| 7 | 24.6 |  | 63 | 24.4 | 31.7 |  | 55 | 21.3 | 5.5 |
